# What’s in a name? Metabolite identification: challenges and pitfalls in untargeted metabolomics

**DOI:** 10.1007/s11306-025-02387-0

**Published:** 2026-02-09

**Authors:** Georgios Theodoridis, Oliver Fiehn, Michal Holčapek, Royston Goodacre, Daniel Raftery, Robert Plumb, Timothy M. D. Ebbels, Michael Witting, Helen Gika, Ian D. Wilson

**Affiliations:** 1 Department of Chemistry, Aristotle University of Thessaloniki, Thessaloniki, 54124 Greece; 2 Biomic AUTh, Center for Interdisciplinary Research and Innovation (CIRI- AUTH), Balkan Center B1.4, 10th km Thessaloniki-Thermi Rd, P.O. Box 8318, Thessaloniki, 57001 Greece; 3 West Coast Metabolomics Center, UC Davis Genome Center, University of California, 451 Health Sciences Drive, Davis, CA USA; 4 Department of Analytical Chemistry, Faculty of Chemical Technology, University of Pardubice, Studentská 573, Pardubice, 53210 Czech Republic; 5 Centre for Metabolomics Research, Department of Biochemistry, Cell and Systems Biology, Institute of Systems, Molecular and Integrative Biology, University of Liverpool, BioSciences Building, Crown St, Liverpool, L69 7ZB UK; 6 Northwest Metabolomics Research Center, Department of Anesthesiology and Pain Medicine, University of Washington, 850 Republican St, Seattle, WA 98109 USA; 7 Waters Corporation, 34 Maple St, Milford, MA USA; 8 Division of Systems Medicine, Department of Metabolism, Digestion and Reproduction, Imperial College London, Hammersmith Campus, London, W12 0NN UK; 9 Metabolomics and Proteomics Core, Helmholtz Zentrum München, Ingolstädter Landstraße 1, 85764 Neuherberg, Germany; 10 Chair of Analytical Food Chemistry, TUM School of Life Sciences, Technical University of Munich, Maximus-von-Imhof-Forum 2, 85354 Freising-Weihenstephan, Germany; 11 Department of Medicine, Aristotle University of Thessaloniki, Thessaloniki, 54124 Greece

**Keywords:** Research integrity, Metabonomics, Biomarkers, Mass spectrometry, Metabolite annotation, Lipidomics

## Abstract

**Background:**

The aim of metabolic phenotyping (metabotyping) is to discover and identify metabolites (including lipids) that can be used to characterize biological samples and differentiate between different physiological states. The identification of the metabolites responsible for this differentiation is essential if mechanistic understanding is to be obtained. Confident metabolite identification arguably represents the most important outcome of untargeted metabolomics studies but currently the standards used for metabolite identification reported in many publications do not strictly follow the various published guidelines and thus these identifications lack sufficient proof.

**Aim of review:**

In this perspective we define problems that currently plague the field of metabolite identification using MS-based techniques, particularly LC-MS, in untargeted metabolic phenotyping. Despite considerable efforts by the community (researchers, instrument manufacturers, software, and database developers) this continues to be a contentious and error-prone step in the metabolomics workflow. The majority of publications provide only sparse data on the evidence for metabolic markers “identified” and we have observed an alarming increase in the frequency of erroneous metabolite identifications. Here, we describe the problem and provide several illustrative case studies. Our goal is to raise awareness and highlight the issue of poor metabolite identification, since it is also increasingly apparent that these errors are not always recognised during the reviewing process, such that papers with potentially erroneous metabolite identities reach publication.

**Key scientific concepts of review:**

Poor metabolite identification potentially represents an existential threat to the credibility of untargeted “discovery” metabolomics and can pollute the literature. Here we describe the aetiology of the problem and explain how and why this issue affects the field. We argue that coordinated action is required by researchers, database managers, scientific societies and the reviewers, editors and publishers of scientific journals to both acknowledge and address this important problem.

**Supplementary Information:**

The online version contains supplementary material available at 10.1007/s11306-025-02387-0.

## Introduction

According to the accepted process for metabolite identification, metabolites described as “identified”, should ideally be identified to Level 1 of the Metabolomics Standards Initiative (MSI) or equivalent schemes (Fiehn et al. 2007, Sumner et al. 2007, Lindon et al. 2005, Schymanski et al. 2014), based on the direct comparison with data from the analysis of authentic standards measured under identical analytical conditions. In a similar manner, identification of lipids should follow the criteria recommended by the Lipidomics Standards Initiative (LSI) (Liebisch et al. 2020). Where Level 1 identification is not possible by LC-MS due to the non-availability of authentic standards, but robust supporting spectroscopic data (e.g., both MS and NMR) provides sufficient information, then it may still be possible to claim a definitive identification. Level 1 identifications can sometimes be ambiguous, e.g., if tryptophan is reported one cannot be sure whether it is the D or L enantiomer (or both) that is present, unless chiral methods are used for differentiation. Lower levels of the MSI are typically referred to as annotations, as room for alternative identities exists, and these should be used with great care, and only as “leads” for further investigation, and not for the construction of mechanistic hypotheses in the absence of other supporting information.

Unfortunately, a common practice is that authors claim to have ‘identified’ compounds simply on the basis of performing a match of their mass spectrometric (MS) data against metabolite structure databases, using some arbitrary level of accurate mass. Databases, online biochemical pathway analysis tools and other open source or vendor metabolomics data processing software connected with these databases may use resources in which the majority of spectra are predicted *in silico*. This can result in annotations that are thus not based on experimental data or are not relevant for the studied organism (e.g., finding drug metabolites in plants). Even in the case that these data are indeed experimental, there is no guarantee that the LC-MS data obtained in one instrument under certain conditions are directly comparable with that obtained on different instruments and conditions when analysing complex real samples (e.g., see Gika et al. 2010). Unfortunately, this fact is often not sufficiently considered by researchers, who do not always perform the necessary scrutiny of their data and uncritically report the outcomes provided by the software and tools as definite metabolite identifications, without following the necessary steps proposed in the guidelines (Fiehn et al. 2007, Sumner et al. 2007, Lindon et al. 2005, Schymanski 2014, Kodra et al. 2021, Liebisch et al. 2020, Alseekh et al. 2021) and the literature. We also observe a growing lack of the application of fundamental physical chemistry or biochemistry and basic-level scrutiny using fundamental knowledge of analytical chemistry practice, i.e., an overreliance on automated outputs without sufficient quality control or reality checks.

A further problem is that the easy and convenient use of fast computational and AI tools is highly tempting. Authors performing systematic analysis of the literature via AI tools may end up spreading mistakes such as erroneous metabolite identifications, thus multiplying their impact. If not picked up in time, these errors in metabolite identification may end up populating databases, generating a fake reality, e.g., on the nature or source of certain molecules. As a result, increasing awareness and the education of researchers on the dangers of potential errors in metabolite identification is becoming even more timely and important.

**Fig. 1:**
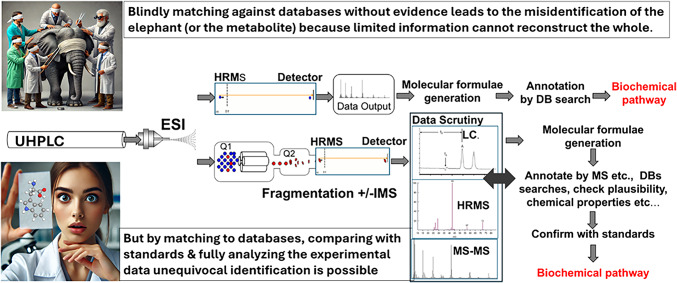
The metabolite identification process is shown as a cartoon for LC-MS(/MS). Current technology can yield both accurate mass (when the MS has accurate resolution and is tuned appropriately) as well as fragmentation patterns for specific ions/metabolites to be annotated. The Figure of the blind men and the elephant, has its origins in India and is a fable about a group of blind men who stumble across a strange creature, and they try to understand what it is (Goodacre, 2019). This is used to represent the analyst who uses shortcuts in metabolite identification. In contrast, the “enlightened scientist” uses appropriate methods for metabolite identification and publishes the evidence base so that it is clear that unequivocal identification has been achieved. UHPLC, ultra-high performance liquid chromatography; ESI, electrospray ionization; HRMS, high resolution mass spectrometry; IMS, ion mobility spectrometry; Q1, quadrupole 1; Q2, quadrupole 2 used for (e.g.) collision-induced dissociation. The Blind Men and the Elephant and the Enlightened Scientist were generated in ChatGPT; the blind men have been kept all male to be consistent with the parable, no gender/diversity related offense is intended by the authors. The molecular structure of tryptophan was generated in MolView (http://molview.org/)

The issue with poor metabolite identification is highlighted in Fig. 1. In this figure the ‘blind’ matching is exemplified by the Indian Parable of the blind men and the elephant. In this parable a group of blind men encounter an unfamiliar creature (think metabolite) and attempt to identify what it is. Each man is of course inaccurate as they only examine a small part of the elephant, and only by sharing their individual experiences would they have understood that this strange creature was an elephant (Goodacre, 2019). This concept can be applied to metabolite identification, in which case those responsible use a battery of methods/data to unequivocally identify the unknown compound e.g., high resolution MS and fragmentation patterns and orthogonal information such as retention time (*t*_R_), collisional cross sections (CCS) and (bio)chemical knowledge etc. To ensure fact-based identification appropriate databases can also be, and are, used to support metabolite annotation along with analysing standards on the same analytical platforms.

As reviewers, editors of scientific journals and active researchers, working on metabolomics, we have become increasingly concerned about the often-poor quality and inadequate rigour applied to metabolite identification in submitted and (unfortunately) also published papers particularly those dependent on MS-based analyses. In particular, we are very concerned that metabolite identification, which is clearly one of the most critical aspects of untargeted metabolomics and central to the biochemical-based hypotheses that emerge from such studies, is not always being performed or reported to an appropriate standard as recently described (Wilson et al., 2021; Theodoridis, et al., 2023). In a typical untargeted study, not all detected features will be identified unequivocally due to resource and other constraints. However, where features are annotated/identified (i.e., associated with a chemical structure), the level of confidence should be reported according to community accepted standards (Sumner et al. 2007, Schymanski et al., 2014). Here we highlight a few examples of poor metabolite annotation/identification that have appeared in the recent literature, indicating some of the collective failures of authors, reviewers and editors in maintaining sufficient standards in metabolite identification. These have been selected as examples of a wider problem and are not intended to cause offense or blame, but rather to serve as a call for the community to do better collectively. We appreciate the difficulty in maintaining these standards and also acknowledge that the field is still under development; in hindsight many earlier studies may exhibit such problems as knowledge is constantly evolving and it takes time to disseminate new standards (hence motivating this article to raise awareness).

### Case studies

Case 1: Article “*Serum metabolic profile and metabolome genome-wide association study in chicken*” (Tian et al. 2023), claims: “*Metabolomics was used to study the serum of 508 12-week-old chickens”; “We constructed a chicken serum metabolite dataset containing 7*,*191 metabolites to provide a reference for future chicken metabolome characterization work*”.

The following problems in metabolite annotation and identification were found.

- First, the data provided to support the identification of the metabolites offers little confidence.
- A very large number of exogenous and xenobiotic compounds are reported in supplementary Table S1 of the article: the term exogenous is reported 6696 times, and the term endogenous 5321 times. The term INN is found 77 times in the table next to a molecule name. INN stands for International Non-proprietary Names to facilitate the identification of pharmaceutical substances or active pharmaceutical ingredients. Similarly, substances are characterised as USP, BAN, JAN, USAN that describe pharmaceutical substances from the US pharmacopoeia and the British, Japanese and US reference systems respectively. Such “hits” should ring alarm bells to authors, reviewers, and editors. A small collection of the pharmaceuticals/pesticides reported in the paper includes artesunate, dapsone, pimozide, flucofuron, prednisolone, and prednisone; Supplementary information provided with this perspective contains a non-exhaustive list (*ca*. 40 examples) of such implausible identifications. We argue that it is highly unlikely that all these drugs are present in the blood of the studied chickens. Further, a large number of natural products are reported. These might be diet-derived, however insufficient proof of identity is provided, for either the drugs or these natural products.
- The analytical platform used by the authors could not provide the structural information for reporting the highest annotation level; e.g., used in the LIPIDMAPS database. Instead, the experimental evidence may support only the lipid species level in the shorthand annotation of lipids (Liebisch et al. 2020), which should have been used instead. The structures are not supported by sufficient evidence, remaining at most tentative (Level 3 or lower) annotations. Moreover, no criteria on library matching were stated, which further confounds any evidence basis for these metabolite annotations.
- For many compounds the chromatographic retention times were not consistent with the proposed identifications. For lipids, many tentative identifications did not follow the logical retention dependencies for lipid classes (Vaňková et al. 2022), which strongly suggests that at least some, if not all, of the tentative identifications were incorrect. In addition, there are numerous chromatographically implausible entries i.e., substances that are practically impossible to detect on RPLC and LC-MS or appear with an unrealistic elution time or elution order. Finally, while relatively trivial, the accuracy of the reported retention times (five digits of a second i.e. 607.07938 s) is meaningless in the context of normal LC retention fluctuations, which is likely in the order of seconds not milliseconds or less.

The Supplementary information provided with this perspective includes lists of questionable identifications categorised into four sections: (1) Exogenous/xenobiotic entities, (2) Chromatographically implausible entries, (3) Unexpected identifications for LC, (4) Overly sophisticated identifications. We have also provided detailed justification for our comments.

In the absence of further supporting information to verify the identifications of the compounds reported, our observations indicate that the whole list cannot be relied upon based on the presented evidence. As a result, we believe that the author’s claims in the abstract and the text on building a “chicken serum metabolite dataset” are not supported by the evidence provided and that, far from being useful as a reference, many are likely to be incorrect and cause further incorrect annotations. If taken at their face value, this dataset may hamper science, because it is possible that other researchers will use these spurious annotations as factual identifications in their own work. Further discussion of this study is included in the Supplementary Information.

Case 2: *Article “Lactobacillus paracasei L9 affects disease progression in experimental autoimmune neuritis by regulating intestinal flora structure and arginine metabolism” (*Meng, et al. 2023*)* claims: *“Faecal metabolomics and 16S microbiome analysis”* the identified markers included: *mitoxantrone, methylprednisolone succinate and, dhas#18.*

The first two entries are synthetic pharmaceuticals, however, mitoxantrone is regarded in the paper as a molecule subject to “down-regulation” or “up-regulation”. In addition the metabolite dhas#18 is a sex pheromone that is produced by males of the sour paste nematode *Panagrellus redivivus*. There are also molecules with irregularly long names. Two such examples are taken from Fig. 6 of the article in question.

(2s, 3r, 5r, 10r, 13r, 14 s, 17 s)-2,3,14-trihydroxy-10,13-dimethyl-17-[(2r,3r)-2,3,6-trihydroxy-6-methylheptan-2-yl]-2,3,4,5,9,11,12,15,16,17-decahydro-1h-cyclopenta[a]phenanthren-6-one.

(2s, 3r, 5r,10r,13r,14s,17s)-2,3,14-trihydroxy-10,13-dimethyl-17-[(2r,3r-)-2,3,6-trihydroxy-5,6-dimethylheptan-2-yl]-2,3,4,5,9,11,12,15,16,17-decahydro-1h-cyclopenta[a]phenanthren-6-one.

Identification of these two structures, with the detailed positions of functional groups and stereochemistry (if possible at all) requires high experimental sophistication and expertise, which is not reported in this article; only “KEGG differential metabolic pathway analysis” was used. Overall, the descriptions of the applied processes are not satisfactory; no mass spectral or chromatographic data are presented. The article claims “1797 metabolites were identified in rat faeces”, however, the process, criteria, and tools are not described sufficiently.

Case 3: *Article “Association of cord plasma metabolites with birth weight: results from metabolomic and lipidomic studies of discovery and validation cohorts”* (Xie et al. 2024) *reports LC-MS based “pseudotargeted metabolomics approach applied in the analysis of 504 cord blood samples”.*

The supplementary table is labelled: “Metabolite-wide association study (MWAS): estimated linear association of 2418 individual metabolites and birth weight for gestational-age Z-score”. The differential metabolites are characterised with *p*-values, confidence intervals and pFDR, but the list includes several synthetic drugs/pesticides such as prasterone enanthate, acetaminophen, acamprosate, azacitidine, phenformin, mupirocin, clavulanic acid, nitrofurazone, mefloquine, taprostene, oxypurinol (a metabolite of synthetic allopurinol), cadusafos, dexrazoxane, mexiletine, fenothiocarb, mezlocillin, etomidate, ropivacaine, venlafaxine, melphalan, phenothrin, quinmerac, and phenthoate. We argue that it is highly unlikely that all these drugs are present in the blood of all (or even a majority) of these subjects. Other implausible “markers” include Chaps (a detergent of laboratory use) and insect hormones such as ecdysone (and related phytoecdysteroids) and insect juvenile hormones as well as industrial chemicals such as 3,5,6-trichloro-2-pyridinol.

Case 4: *Article “Multi-omics analysis of magnetically levitated plasma biomolecules”* (Ashkarran et al. 2022*) claims: “we demonstrate that there are particular lipids and metabolites in various layers of each specific plasma pattern that significantly contribute to the discrimination of different multiple sclerosis subtypes”.*

The list of 382 identified metabolites includes synthetic drugs such as pitavastatin, rizatriptan, clotiazepam, moxalactam, pronetalol hydrochloride, chlorpromazine, dexchlorpheniramine, zomepirac. **c**eforanide, ketazolam, talampicillin along with the fungicide diclomezine and the herbicides pyriftalid, metolachlor, delachlor, alachlor and acetochlor as well as the dye disperse yellow and numerous other synthetic chemicals (*ca*. 90 in total).

Case 5: *Article “Immune and metabolic effects of African heritage diets versus Western diets in men: a randomized controlled trial” (*Temba et al. 2025*) aims to study how the switch from heritage-style to Western-style diet affects different metabolic pathways in human males.*

Flow injection MS/MS provided “1,266 ions and 1,339 molecular formulas, with 935 metabolites annotated in the Human Metabolome Database (HMDB)”. Then pathway analysis was performed to “identify metabolites within or influencing specific pathways”. However, Supplementary Table S10 titled “Differential analysis results metabolites” contains numerous synthetic drugs, pesticides and chemicals such as: Dacarbazine, temozolomide, nitrofurazone, monobenzone, diethylpropion, 1-naphthalenesulfonic acid, aprobarbital, butalbital, pentobarbital sodium, gemfibrozil, mizoribine, oxprenolol, tazobactam, duloxetine, quinalphos, ethiofencarb, Triton X 100, pemirolast, acetaminophen glucuronide, and paracetamol sulfate. The authors state that "Drugs that were detected using manual curating in the downstream analysis were flagged in the summary table and were disregarded in the analysis" with pathway analysis performed to "identify metabolites withinn orinfluencing specific pathways". However, there is little information in the manuscript in support of any of the identifications and it is e.g., difficult to see how MS/MS alone can provide confidence for complex structure such as (2Z,4"Z)-2-(5-Methylthio-4-penten-2ynylidene)-1,6-dioxspiro[4.4]non-3-ene or TG(22:4(7Z,10Z,13Z,16Z)/18:2(9Z,12Z)/22:5(7Z,10Z,13Z,16Z,19Z)).

Case 6: *Article “Integrating Metabolomics and Genomics to Uncover Antimicrobial Compounds in Lactiplantibacillus plantarum UTNGt2*,* a Cacao-Originating Probiotic from Ecuador”* (Molina et al. 2025) *analyzed intracellular and extracellular metabolites of Lactiplantibacillus plantarum UTNGt2 to identify molecules associated with antimicrobial activity.*

The tables of detected metabolites included synthetic drugs such as glimepiride, pravastatin, loperamide, chemicals such as dibutylphthalate and with functional group positions reporting at a level of detail not supported by the data e.g.: 2-[1-[1-hydroxy-10,13-dimethyl-3-[3,4,5-trihydroxy-6-[[3,4,5-trihydroxy-6-(hydroxymethyl)oxan-2-yl] oxymethyl]oxan-2-yl]oxy-2,3,4,7,8,9,11,12,14,15,16,17-dodecahydro-1H-cyclopenta[a]-phenanthren-17-yl]ethyl]-4,5-dimethyl-2,3-dihydropyran-6-one.

Case 7: *Article “Different software processing affects the peak picking and metabolic pathway recognition of metabolomics data”*(Liao et al. 2022) claim: *“the effects of different chromatographic columns and software pretreatments on metabolomics data were evaluated based on clinical large cohort samples*,* which will provide a reference for the metabolomics of clinical samples and guide subsequent mechanistic research”.* Further-on the claim is made that: *“This study may help researchers critically explore the distinct alternatives for LC-MS metabolomics data analysis to better choose the most appropriate software”.*

In this work different data mining software were evaluated, and the paper contains a section titled “*Identified differential metabolite comparison of different software and columns*”. However, despite such claims for focus on analytical quality and on critical reviewing the data, the reported “differential metabolites” include a large number of synthetic drugs or their metabolites such as e.g.: Imidaprilat, carisoprodol, methylprednisolone, butalbital, etretinate, secobarbital, phenmetrazine, fluvastatin, desflurane, zoledronate, paracetamol sulfate, letrozole, azlocillin, azatadine, levetiracetam, zileuton, treosulfan, riluzole, dacarbazine, sulfaphenazole, valganciclovir, candoxatril, metrizamide, desmethyl-mirtazapine, mestranol, hydroxy-clobazam, ethosuximide, sulfamethoxazole and saxagliptin. As well as these drug substances further implausible identities included silica, butyric acid (the separation was performed on a C18 column) and hydrogen carbonate, an inorganic substance.

In all these cases we argue that it is highly unlikely that all of these drugs were present simultaneously *in all (or even a majority) of the studied organisms.* Further, simply removing the obviously implausible compounds does not mean that the remaining identifications are reliable. Whilst these drugs have legitimately been reported in clinical samples (e.g. after administration of the specific drug) their detection in discovery studies should be confirmed by analysis of authentic standards. Importantly the incidence of them in patient samples should have been reported and justified. Claiming the presence of multiple drugs in samples where they would not be expected warrants the highest level of analytical scrutiny. Critically, there was little, if any attempt in these papers to report the confidence level of the annotations according to one or more of the community-accepted standards. Most importantly, and perhaps rather unfortunately, the same reservations apply to the pages of biochemistry and biomarker discovery discussion provided in these papers.

## Do’s and don’ts in metabolite annotation

The papers we have discussed in this perspective are not untypical, and were not singled out for special criticism, but came to our attention while searching and reading the relevant literature. They are presented as examples of how unverified annotations are appearing in the literature. Indeed, they are typical of many of the manuscripts that are submitted to metabolomics-oriented journals where, thanks to expert Reviewers and Editors, many of these errors are corrected (although these defences are not infallible). The major problems however, probably arise where metabolomics research is submitted to journals with little focus on, or expertise in, this area at both reviewer and editorial level. In such cases poor decisions are much more likely to be made. In preparing tables of annotations, as opposed to Level 1 or 2 identifications, especially where the suggested structures are derived largely from database derived annotations the do’s and don’ts provided in Table 1 may be worth considering.

**Table 1:** Do’s and don’ts in metabolite Annotation/Identification and reporting for LC-MS-based studies

**Don’t** blindly trust automated software	**Do** validate outputs and question strange identifications
**Don’t** report the same metabolites with different *t*_R_ in the same chromatogram	**Do** reality check your that *t*_R_ data are consistent with proposed structures
**Don’t** use signals from fragments in the “noise”	**Do** provide evidence of the signal/noise ratio level used for fragment selection
**Don’t** report stereochemistry without evidence	**Do** think about the level of knowledge available and the technology used (e.g., if achiral separation was used etc.)
**Don’t** trust matches based on e.g., database searches only on single ions without further evidence	**Do** consider similarity, fragment match etc., and consider reporting a fragment match or similarity score.
**Don’t** ignore evidence that does not support “identifications”	**Do** use/obtain “orthogonal” data other including *t*_R_, and migration time etc., from separations (CE, GC, SFC, IM), LogP calculations, NMR spectroscopy, etc. where possible to support identifications
**Don’t** report exogenous/or xenobiotic compounds blindly	**Do** verify if medication/exposure to xenobiotics is expected in the study context
**Don’t** provide annotations/identifications without considering the levels of annotation confidence	**Do** report a level of confidence using community accepted standards for *all* annotations
**Don’t** provide lists of un-curated annotations	**Do** show all the data for metabolites used in hypotheses

If such careful data “curation” was widely adopted the prevalence of erroneous metabolite identification would be greatly reduced. The wealth of information (e.g., *t*_R_, CCS values, product ion spectra) collected by modern instrumentation is often underexplored. If authors also followed the widely available guidance of bodies such as the Metabolomics Society and the International Lipidomics Society etc., and research groups for the annotation, identification and appropriate reporting of compounds detected in untargeted studies, data quality would be greatly improved. It is also the responsibility of reviewers and editors to ensure that this evidence is provided before or during the review process. As an aid to improving the situation, we would draw the reader’s attention to the resources available that have been published defining the metabolomic and lipidomic content of multiple matrices, e.g. in accordance with the current consensus of quality requirements for LC-HRMS lipidomics data, the NIST^®^ Standard Reference Material for Human Plasma (SRM 1950) was recently characterised by LC-MS (Martínez et al. 2024, 2025). A highly curated lipid database with increased coverage, quality and consistency was generated, including quality assurance procedures that included adduct formation, within-method *m/z* evaluation, retention behaviour of species within lipid chain isomers, and expert-driven resolution of isomeric and isobaric interferences. Likewise, many databases have been constructed for specific organisms or matrices, e.g. for the human faecal metabolome (https://fecalmetabolome.ca/) or others. Such reference databases can be used for plausibility checks of annotations and identifications, and we would encourage potential authors and reviewers to use them to help towards obtaining fact-based metabolite annotations. Additionally, some metabolite and lipid structure databases, such as ChEBI or LIPIDMAPS include taxonomic information for additional interpretation. Furthermore, they contain shorthand notations and identifiers for partial structures in case the full structure cannot be identified,.e.g., when working with lipids (Witting et al. 2024).The collaboration of researchers, database providers, instrument vendors and other stakeholders toward the generation of community accepted standards (that could include schemes such as traffic-light classification on metabolite identification) could prove very useful towards achieving these ends. Lastly, we would urge those performing metabolic phenotyping using MS-based techniques such as LC-MS not to become reliant on the MS data obtained as the sole basis for metabolite identification. Indeed, the need for orthogonal information in order to achieve the highest possible levels of confidence in metabolite identification guidance was clear even from the earliest publications concerned with the reporting these data (Lindon et al. 2005, Fiehn et al. 2007).

## Conclusion

Incorrect metabolite identification and erroneous peak annotations have the potential to damage the credibility of metabolic phenotyping (metabolomics, metabonomics, metabotyping) and the incidence of these problems must be reduced, even if they cannot be eliminated altogether. We believe that the views expressed here are shared by many in both the established metabolomics community and relevant associated societies. Interested researchers are encouraged to take action on this issue, individually and collectively. If this is not done the literature will continue to amass large amounts of questionable data. In an age when AI is increasingly used to scan through large amounts of data, the potential for it to return nonsense as metabolomic facts should not be underestimated and could undermine the large amount of high-quality knowledge that has been amassed by the community over many years.

## Supplementary Information

Below is the link to the electronic supplementary material.

Supplementary Material 1

## Data Availability

No datasets were generated or analysed during the current study.
