# Supplementary material for: What’s in a name? Metabolite identification: challenges and pitfalls in untargeted metabolomics
Source: Metabolomics. 2026 Feb 9;22(2):22. doi: 10.1007/s11306-025-02387-0 (PMC12886295; doi:10.1007/s11306-025-02387-0)
Supplement: Supplementary file 1 — Supplementary Material 1 [file 11306_2025_2387_MOESM1_ESM.docx]

**Supplementary Information**

**What’s in a Name? Metabolite Identification Challenges and Pitfalls in Untargeted Metabolomics.**

Georgios Theodoridis^1,2^, Oliver Fiehn^3^, Michal Holčapek^4^, Royston Goodacre^5^, Daniel Raftery^6^, Robert Plumb^7^, Timothy M D Ebbels^8^, Michael Witting^9,10^, Helen Gika^2,11^, Ian D Wilson^5,12*^

^1^ Department of Chemistry, Aristotle University of Thessaloniki, Thessaloniki 54124, Greece

^2^ Biomic AUTh, Center for Interdisciplinary Research and Innovation (CIRI-AUTH), Balkan Center B1.4, 10th km Thessaloniki-Thermi Rd., P.O. Box 8318, Thessaloniki 57001 Greece

^3^ West Coast Metabolomics Center, UC Davis Genome Center, University of California, 451 Health Sciences Drive, Davis, CA, USA

^4^ Department of Analytical Chemistry, Faculty of Chemical Technology, University of Pardubice, Studentská 573, 53210 Pardubice, Czech Republic

^5^ Centre for Metabolomics Research, Department of Biochemistry, Cell and Systems Biology, Institute of Systems, Molecular and Integrative Biology, University of Liverpool, BioSciences Building, Crown St., Liverpool, L69 7ZB, United Kingdom

^6^ Northwest Metabolomics Research Center, Department of Anesthesiology and Pain Medicine, University of Washington, 850 Republican St., Seattle, Washington 98109, United States

^7^ Waters Corporation, 34 Maple St, Massachusetts, USA

^8^ Division of Systems Medicine, Department of Metabolism, Digestion and Reproduction, Imperial College London, Hammersmith Campus, London W12 0NN, United Kingdom

^9^ Metabolomics and Proteomics Core, Helmholtz Zentrum München, Ingolstädter Landstraße 1, 85764 Neuherberg, Germany

^10^ Chair of Analytical Food Chemistry, TUM School of Life Sciences, Technical University of Munich, Maximus-von-Imhof-Forum 2, 85354 Freising-Weihenstephan, Germany

^11^ Department of Medicine, Aristotle University of Thessaloniki, Thessaloniki 54124, Greece

^12^ Division of Systems Medicine, Department of Metabolism, Digestion and Reproduction, Imperial College London, Hammersmith Campus, London W12 0NN, United Kingdom

*** Corresponding author** (i.wilson@imperial.ac.uk)

As discussed in the main text, metabolite identification represents a major challenge in metabolomics and lipidomics with emphasis on the following requirements: Authors should provide all data required by the Metabolomics Standards Initiative (MSI) of the Metabolomics Society [1], which includes a combination of RT, MS data, MS/MS data, NMR spectroscopy data etc. in accordance to FDA, EMA guidelines such as the EU Committee 657, 2002 guidance document for analytical laboratories. For the identification and reporting of lipids, the recommendations of the Lipidomics Standards Initiative (LSI) of the International Lipidomics Society (ILS) should be followed [2][Harald C. Köfeler et al, J. Lipid Res. (2021) 62 100138].

We have chosen to explore one manuscript in more detail. The highlighted issues are common to many publications. We wish again to emphasize that this is a general problem and not specific to this particular study.

In their work as discussed here, the authors claim that they identified >7000 analytes in the serum of chickens. Unfortunately, the real experimental data provided in support of such claims is meagre, so a large number of identification claims are not proven.

Some of the authors of this perspective have recently published two editorial notes on this problem [3-4]. In these articles we highlighted two major categories of common errors.

1. Reporting xenobiotic analytes unrelated to the specific specimen. Such claims need strong additional evidence.

2. Reporting analytes with physicochemical properties not compatible with the reported data (especially the case for chromatographic separations).

The manuscript in question provides a wealth of examples in both categories. We highlight below a small number of such examples of data and metabolite identification, provided by them that are in our opinion not substantiated to the required level. It is very likely that there are more such cases in these data.

**Section 1. Exogenous / Xenobiotic entities**

Regarding the plausibility of the proposed identifications, of Tian et al. (supplementary information **Table S1**) contains more than 2300 entities (correctly) characterized by the authors as exogenous substances [5]. We believe that there are many chemicals, particularly pharmaceuticals, supposedly present in control chicken serum that are very unlikely and such claims require verification ideally to level 1 of the MSI . Reporting the detection of such analytes should be justified and should be supported by additional strong evidence (which usually consists of two orthogonal chemical properties - such as retention time and accurate mass plus MS/MS data - matched to an authentic standard). In the present case a very large number of pharmaceuticals and pesticides are claimed as present which, if true, would be a cause of some concern to consumers. Even if the chickens were treated with veterinary medicines or exposed via feedstock, most of these annotations remain implausible.

Some examples of such unproven claims are highlighted in red font in the attached table indicating a large number of analytes that are suspect identifications representing molecules that need meticulous scrutiny and strong evidence. We do not claim that this is a comprehensive listing of unlikely identifications, these are simply the most obvious examples. Further to our original check we searched for these molecules in chemical and pharmaceutical databases (PubChem, ChemSpider, the IUPHAR/BPS Guide to PHARMACOLOGY) to ensure that a compound was of natural origin or not. We believe that if a xenobiotic (drug, pesticide etc.,) is proven to be in the serum of control chickens its presence should be explained. A large number of exogenous natural products were also “identified” in the Table. While the presence of natural products may be plausible for some of them, for example as being part of the feeding regime, reporting such a large number of natural toxins, alkaloids, secondary metabolites etc. in the blood of farmed animals should ring alarm bells and should have been investigated further and justified. We have highlighted analytes below which are entirely synthetic in origin. A selection of these xenobiotics claimed as being present in the serum of chickens is given below along with a short description (further examples are highlighted in the attached table):

1. Astemizole is a synthetic piperidinyl-benzimidazole derivative

2. Betamethasone Valerate is the 17-valerate ester of betamethasone, a synthetic glucocorticoid

3. Oxandrolone is a synthetic anabolic steroid.

4. Valganciclovir is a prodrug of ganciclovir, an approved antiviral drug. If administered it should be transformed to ganciclovir.

5. Amidosulfuron is a broad spectrum herbicide

6. Celecoxib is a non-steroidal anti-inflammatory drug (NSAID)

7. Thiomersal drug developed and registered under the trade name Merthiolate by Eli Lilly and Company.

8. Artesunate is a semisynthetic derivative of artemisinin.

9. Dapsone is a sulfonamide-related antibacterial, antimalarial and anti-inflammatory drug.

10. Gemfibrozil is a fibric acid antilipemic agent, pharmacologically similar to clofibrate.

11. Pimozide is an antipsychotic drug of the diphenylbutylpiperidine class.

12. Delavirdine is an antiretroviral agent that acts by noncompetitive binding to and inhibition of the HIV reverse transcriptase

13. Udenafil (Zydena) is a PDE5I developed by a pharmaceutical company in 2005

14. Perindoprilat is a metabolite of perindopril, an anti-hypertension synthetic drug.

15. PerindoprilErbumine is a prodrug, that means that even if the farmers have indeed delivered the drug formulation for hypertension to the hens, this molecule would hydrolyze in the blood to form Perindoprilat.

16. Lisinopril and fosinopril are also anti-hypertension synthetic drugs of the same category as the above. How can it be that chicken blood contains three such drugs?

17. Promazine is a phenothiazine antipsychotic.

18. Prednisolone and Prednisone are synthetic glucocorticoids.

19. Desogestrel is a progestin (synthetic progesterone-like agent)

20. Megestrol (commonly administered as the acetate ester is a synthetic derivative of the female sex hormone progesterone.

21. Alclometasone is a synthetic corticosteroid for topical dermatologic use.

22. Flucofuron is an organofluorine pesticide

23. Otenzepad is a competitive muscarinic receptor antagonist that is relatively selective at the M2 receptor. It was investigated as a treatment for arrhythmia.

24. Chloramphenicol is a semisynthetic, broad-spectrum antibacterial

25. Irbesartan is an angiotensin receptor antagonist.

26. Mifepristone is a drug used to block progesterone in order to end a pregnancy.

27. Doxycycline is a tetracycline based antibacterial.

28. Delavirdine is a non-nucleoside reverse transcriptase inhibitor (NNRTI) used to treat HIV-1

29. Carbamazepine is a tricyclic anticonvulsant.

30. Practolol is a selective β-blocker

31. Atenolol is a selective β1-adrenoceptor antagonist (β blocker)

32. Promethazine is a first-generation antihistamine pharmaceutical.

33. Tafluprost is a prostaglandin analogue.

34. Boldenone is an anabolic androgenic steroid and synthetic derivative of testosterone

35. Buspirone is an anxiolytic psychotropic drug.

36. Barbiturates (synthetic drugs very well known to the common public) are reported 12 times in Table S1.

**Section 2. Chromatographically Implausible Entries**

General comment: Reporting of retention in liquid chromatography in seconds is not normal practice (generally minutes are used) and the accuracy of the reported retention times (five digits of a second i.e. 607.07938 sec) is meaningless with the accuracy of the LC methods reported (fluctuations in the first two decimal places of RTs given in minutes is expected).

**Tryptophan**

Line 10 Dl-Tryptophan with retention time 1666.06304 sec.

Line 12 D(+)-Tryptophan with retention time 607.07938 sec.

a. The use of the prefixes “DL” and “D” presumably refer to the optical enantiomers of tryptophan. However, if a chiral separation was indeed used it is not described in the experimental section. Conventional LC separations do not resolve enantiomers and no supporting information is provided to support these identifications.

b. From the Supplementary **Table S1** provided by the authors with their manuscript it would appear that the authors first report the detection of the unresolved DL racemic mixture in line 1 followed by the D enantiomer alone on line 12. This result is not possible.

c. The retention times are reported to five decimal places of accuracy, which is not achievable with the LC separation used. When reported in seconds, a maximum of one decimal place would be appropriate, or even integer digits.

**Alanine**

Lines 1864, 1996, 3654 and 5779 all report the analyte as alanine, a well-known amino acid. The retention times are 196, 960, 641 and 74 seconds respectively (again with 5 digits accuracy). Lines 1864 and 5779 report use of a T3 column (RPLC). Lines 1996 and 3654 report use of a HILIC column. It is unclear why one molecule appears at two very different retention times (196 and 74 sec on HILIC, 960 and 641 sec on RP) when the same column, and thus same chemistry is used for separation. This should be a cause for concern and clear explanation. Given that alanine is readily available as a standard these annotations could (and indeed should) have been confirmed to level 1 of the MSI, at which stage it would be clear which, if either, of the annotations were correct per column.

Glucose

Lines 1734 and 3792 report detection of glucose on T3 and HILIC columns with corresponding retention times 1580 and 150 seconds, respectively. This result is in contrast to well established data and the experience of the chromatographic community that is explained by the physicochemical properties of the analytes and the chromatographic theory that governs such separations that dictates that glucose should be better (more strongly) retained on the HILIC column. Again, glucose (more accurately D-glucose) is readily available as a standard and as such this should have been confirmed to level 1 of the MSI.

Further examples

1. Lines 1416 and 1418 report 2-hydroxy-dAMP and 3'-AMP with retention times of 1559.8637 and 1628.89768 sec, respectively, on the T3 column, an RPLC phase. Line 1296 reports progesterone with retention time 1666.68878 sec, line 14 reports cholic acid at 1421.88072 sec. AMP is very polar while progesterone and cholic acid are very apolar. Such molecules cannot elute at similar retention times, nor is it likely that apolar molecules elute before the polar molecules on RPLC. This goes against fundamental knowledge on the physicochemical basis of chromatography and against the experience of practitioners of liquid chromatography.

2. Lines 1884-1886 report 8-hydroxypromazine eluting at 7.22098 sec, 4-hydroxypromazine at 1613.21948 sec, and 3-hydroxypromazine at 1650.44432 sec. These are unrealistic retention time differences between the three analogues.

3. Lines 3244-3246 report the following compounds and retention times: (E,E)-2,4-octadienal (893.2629 sec), (E,E)-2,6-octadienal (954.97398 sec) and (E,Z)-2,4-heptadienal (898.65374 sec). The level of identification sophistication should be justified with data. Furthermore, the elution order is unrealistic. Heptadienal has a shorter carbon chain than the others and should be eluted before the octadienals in RPLC.

4. Further down in line 3255 is: (S)-Malate 2134.56458 sec. There is no case for this elution order as malic acid is very polar and should elute before both octadienal and heptadienal, certainly not with more than double retention time compared to them.

5. Lines 5216 and 5218 report testosterone at 1321.00939 sec and testosterone glucuronide at 1397.23774. Basic chromatographic knowledge and experience would lead to the expectation that the glucuronide elutes before the parent compound in RPLC as it is more polar. The same applies to Cholesterol (1411.34714 sec) and cholesterol glucuronide (2120.74552 sec).

6. Similarly, lines 5217 and 5219 report testosterone decanoate at 1495.00888 sec and testosterone propionate at 1802.63524 sec. The decanoate ester is more lipophilic than the propionate and should elute later.

**Section 3. Unexpected Identifications for LC-MS**

1. Line 1376: HSO3-; Line 1394: Sulfate; Line 1395: phosphoric acid; Line 2717, iodide; Line 5228: thioacetate. These substances are barely retained and thus are really not detectable in RPLC.

2. Line 3180. Thiourea is very hard to detect in LC-MS. A retention time of 6 sec is not really feasible.

3. Line 1205 identifies the analyte as “Acid”. This is not a proper identification.

**Section 4. Overly Sophisticated Lipid Identifications**

This issue of strong overreporting occurred for many lipid identifications, while the experimental data show the proof for the lipid species level or maybe down to the fatty acyl level. There are so many examples of overreporting that it is impossible to list all of them, two examples are shown below.

1. Lines 4126-4144 report sophisticated structure annotations such as: DG(13:0/20:5(5Z,8Z,11Z,14Z,17Z)/0:0)[iso2]. Such sophistication requires clear evidence and data to support the claim.

2. (4Z,7Z,10Z,13Z,16Z,19Z)-Docosahexaenoic acid ethyl ester and such structures are reported throughout the Table. Such level of sophistication in terms of lipid structure requires data to support the classification.

Overall, in the data provided there are dozens of points that appear difficult to accept without justification and need confirmation by real experimental data from the analysis of the reference standard.

**References**

[1] Sumner, L. W., et al. (2007) Proposed minimum reporting standards for chemical analysis Chemical Analysis Working Group (CAWG) Metabolomics Standards Initiative (MSI). Metabolomics, 3, 211– 221, http//doi.org 10.1007/s11306-007-0082-2

[2] Köfeler H.C., et al. (2021), Recommendations for good practice in MS-based lipidomicsJ. Lipid Res. 62 100138] 100138. http//doi.org: 10.1016/j.jlr.2021.100138.

[3] Theodoridis, et al (2023). Ensuring fact-based metabolite identification in LC-MS-based metabolomics. *Analytical Chem.*, *95*, 3909-3916. <https://doi.org/10.1021/acs.analchem.2c05192>

[4] Wilson, I. D., Theodoridis, G., & Virgiliou, C. A. (2021) A perspective on the standards describing mass spectrometry-based metabolic phenotyping (metabolomics/metabonomics) studies in publications. *J. Chromatogr. B*. *1164*, 122515, http//doi.org/10.1016/j.jchromb.2020.122515

[5] Tian, J., Zhu, X., Wu, H., Wang, Y. & Hu, X. (2023). Serum metabolic profile and metabolome genome-wide association study in chicken. *J. Animal Sci. Biotechnol*. *14*, .69. <https://doi.org/10.1186/s40104-023-00868-7>.
